# MXene catalytic amplification-fluorescence/absorption dimode aptamer sensor for the detection of trace Pb^2+^ in milk

**DOI:** 10.3389/fnut.2022.1008620

**Published:** 2022-10-18

**Authors:** Shengfu Zhi, Qi Wei, Chi Zhang, Chenguang Yi, Chongning Li, Zhiliang Jiang

**Affiliations:** ^1^School of Public Health, Guilin Medical University, Guilin, China; ^2^Guangxi Key Laboratory of Environmental Pollution Control Theory and Technology, Guilin, China

**Keywords:** MXene, aptamer, TMB reaction, fluorescence/absorption, Pb^2+^

## Abstract

Lead ion (Pb^2+^) is a toxic heavy metal, which is very harmful to organisms. Therefore, the establishment of a rapid, simple, and sensitive method is of great significance to food safety and human health. It was found that MXeneTi_3_C_2_ nanosheet (NS) has a strong catalytic effect on the oxidation of 3,3,5,5-tetramethylbenzidine (TMB) *via* H_2_O_2_ to form the oxidized product (TMB_OX_); it has a strong fluorescence peak at 415 nm and an absorption (Abs) peak at 295 nm. The aptamer of Pb^2+^ (Apt_pb_) can be adsorbed on the surface of an NS to form MXene-Apt conjugates, which reduces its catalytic active sites and inhibits its catalytic activity. When the target Pb^2+^ is added, it specifically binds with Apt_pb_ to release MXene NSs to enhance the dimode signals. Therefore, a new MXene catalytic fluorescence/absorption dimode aptamer biosenering platform was fabricated for the determination of trace Pb^2+^ in milk and water samples, with the fluorescence assay linear range (LR) of 5.0 × 10^−2^-2.0 nmol/L.

## Introduction

MXene is a new two-dimensional (2D) nanomaterial with excellent physicochemical properties and has been used in energy storage, catalysis, tribology, antibacterial, and electromagnetic shielding (1). In analytical chemistry, based on its excellent fluorescence properties, fluorescent probes of Mxene were constructed. Kong et al. (2) synthesized MXene-like quantum dots TiCNQDs using a simple hydrothermal procedure, which exhibited excellent fluorescence properties for the specific and sensitive detection of iron ions with a linear range (LR) of 2-400 and 500-800 μmol/L, respectively, and a minimum detection limit of 1.0 μmol/L. Guan et al. (3) prepared nitrogen- and phosphorus-functionalized Ti_3_C_2_MXene-based quantum dots (N, P-MQDs) using a top-down hydrothermal procedure, which produced green fluorescence at specific wavelengths and exhibited excellent photostability and pH resistance ability. It was proven that N, P-MQDs could be used as fluorescent probes labeled with macrophages and showed the advantages of low cost and high sensitivity in the detection of copper ions. Desai et al. (4) provided a new exploration for selective sensing of metal ions (Ag^+^ and Mn^2+^) *via* fluorescence quenching of Ti_3_C_2_-MXene nanosheets (NSs). The MXene-Ti_3_C_2_ NS monolayer exhibited an excellent ability to sense Ag^+^ and Mn^2+^ ions due to its good hydrophilicity and unique surface functionality. The method was used for the detection of Ag^+^ and Mn^2+^ ions in food and water samples with a high recovery rate. Cui et al. (5) established a specific and high sensitivity fluorescence resonance energy transfer aptamer (Apt) sensor using a single-layer MXene NS, which could be applied for the detection of thrombin and has an important practical significance in clinical diagnosis. As far as we know, MXene has not been used to catalyze the reaction of H_2_O_2_ oxidation of TMB, and combining Apt to build a fluorescence/absorption sensing platform for the analysis of lead ion (Pb^2+^).

Apt refers to a single-stranded deoxyribonucleic acid (DNA) or ribonucleic acid (RNA) nucleic acid molecule with a specific recognition function. Nucleic acid molecules can be folded into a specific three-dimensional spatial structure. Apt can bind to the target molecule with high specificity and high affinity through the spatial structure matching and molecular interaction force between it and the target molecule (6). Compared with other biorecognition molecules, such as antibodies, Apt has a higher specific affinity and can be synthesized in large quantities *in vitro* by polymerase chain reaction (PCR) amplification technology. Several advantages of Apt make it an important research tool in the field of analytical chemistry. Thus, it can be selected as a recognition unit in this study. Fluorescence spectroscopy was a simple and sensitive molecular spectral technique. By combining Apt with fluorescence spectroscopy, some simple, fast, and sensitive Apt fluorescence methods were developed (7–11). Sun et al. (7) developed an upconversing fluorescence DNA probe for the detection of acetamiprid, which consisted of aptamer-conjugated magnetic nanoparticles (Apt-MNPs) and complementary DNA-conjugated upconverting nanoparticles (cDNA-UCNPs). Acetamiprid could specifically bind to Apt-MNPs to separate cDNA-UCNPs from Apt-MNPs and could use an external magnet to reduce fluorescence intensity. The change in fluorescence intensity was positively correlated with the concentration of acetamiprid, and the linear detection range and detection limit were 0.89–114.18 and 0.65 μg/L, respectively. Wang et al. (8) proposed a novel fluorescent Apt sensor for the detection of glypican-3 using glutathione@graphene quantum dot-labeled glypican-3 Apt as a fluorescent probe. When the concentration of glypican-3 was from 5.0 to 150.0 ng/ml, the recovery of fluorescence showed a good linear relationship with the concentration of glypican-3, and the detection limit was 2.395 ng/ml. Shan et al. (9) proposed a highly sensitive fluorescence detection method for okadaic acid in seafood, with a LR of 1.0 ng/L to 50.0 μg/L and a detection limit of 1.1 ng/L. Huang et al. (12) proposed a sensor for the detection of Pb^2+^. Using graphene oxide (GO) as a quencher and an Apt solution as a reagent, the presence of Pb^2+^ ions was detected by measuring the change in the fluorescence intensity of the GO/Apt suspension when the Apt molecule underwent fluorescence resonance energy transfer. Pb^2+^ concentration exhibited a good linear relationship in the range between 10 and 100 nm, and the detection limit of Pb^2+^ was 0.53 ppb. Lei et al. (13) constructed a novel fluorescent method for the detection of Pb^2+^ using CDs/AuNCs ratiometric fluorescent probes. Due to a highly selective fluorescence enhancement effect of Pb^2+^ on CD/AuNC fluorescent probes, the concentration of Pb^2+^ showed a good linear relationship in the range of 20-700 nM. The dimode molecular spectral method not only has the advantages of the single-mode method (14, 15) but also can overcome the disadvantages of the single-mode method. In addition, two modes are available for us to choose from. Thus, the analysis of these modes was shown to be interesting. Based on the nanocatalytic amplification and specific Apt, a surface-enhanced Raman scattering/resonance Rayleigh scattering (RRS) dimode assay was proposed for the detection of trace glyphosate (16). The fluorescence, coupled with the Abs method, to establish a dimode Apt method has the advantages of selectivity, sensitivity, and low cost. A fluorimetric/colorimetric dimode sensing strategy was developed for human papillomaviruses (HPV) DNA, based on the integration of fluorescent DNA-silver nanoclusters and G-quadruplex/hemin DNAzyme (17). Thus, it was significant to fabricate fluorescence/Abs dimode Apt sensors for Pb^2+^.

Lead ion is a heavy metal pollutant with a wide accumulation and distribution. With extensive application and random disposal of lead metal products, it is ubiquitous in the hydrosphere, biosphere, and lithosphere. Because the nervous systems of children are immature, children are more sensitive to Pb^2+^ pollutants, making children a high-risk group for lead poisoning (18). Pb^2+^ poisoning can lead to various diseases, such as blood diseases, liver damage, and kidney damage (19). Therefore, the establishment of a simple, low cost, selective, and sensitive method for the detection of Pb^2+^ has a great practical significance to food safety and human health. In recent years, many researchers proposed different methods, such as inductively coupled plasma optical emission spectrometry or mass spectrometry, high-performance liquid chromatography, and hydride generation atomic fluorescence spectrometry, for the determination of Pb^2+^ (20, 21). To meet the increasing detection demand, many methods that outperform the previous technology have also been developed. Among them, the method developed based on the fluorescence method has the advantages of high sensitivity and low detection limit. Chen et al. (22) developed a colorimetric, label-free, and non-aggregating gold nanoparticle (AuNPs) probe for the detection of Pb^2+^ in an aqueous solution based on the fact that Pb^2+^ ions accelerate thiosulfate leaching of AuNPs with a linear detection range of 2.5 nM to 10 μM, which showed strong practicality in real samples. Cheng et al. (23) developed a simple and selective Pb^2+^ fluorescent probe based on silver sulfide (Ag_2_S) quantum dots. In the presence of Pb^2+^, the coordination between Pb^2+^ and glutathione ligands modified on Ag_2_S QDs could enhance the signal intensity of Ag_2_S QDs through aggregation-induced enhanced emission. The linear detection range of Pb^2+^ was 0.05-20.0 μM, and the detection limit was as low as 15.5 nM. In this study, based on the MXene nanosol-catalyzed H_2_O_2_-TMB reaction to generate TMB_OX_ with a fluorescence/absorption signal in combination with the specific Apt reaction of Pb^2+^, a fast and simple fluorescence/absorption sensing platform for analyzing Pb^2+^ was constructed.

## Experimental section

### Instrument

Fluorescence spectra were obtained by scanning with a Fluorescence spectrophotometer F-7000 (Hitachi High-Technology Corporation. Tokyo, Japan), at a voltage of 400 V, an excited slit and emission slit of 5 nm; TU-1901 Double-beam UV-Vis Spectrophotometer (Beijing Puxi General Instrument Co., Ltd., Beijing, China); and JUPITER-B microwave digestion apparatus (2,200 W, working frequency 50 Hz, Shanghai Xinyi Microwave Chemical Technology Co., Ltd., Shanghai, China); scanning electron microscope images and energy dispersive spectroscopy were taken using the JSM-6380LV field scanning electron microscope (Hitachi of Japan, Tokyo, Japan). Nano-2S nanoparticle size and zeta potential analyzer (Malvern Co., Malvern, UK); HH-S2 electric heating constant temperature water bath (Jintan Dadi Automation Instrument Factory, Jiangsu, China); high-speed refrigerated centrifuge (GL-25MS, Shanghai Luxiangyi Centrifuge Instrument Co., Ltd., Shanghai, China); KQ3200DB CNC ultrasonic cleaner (Kunshan Ultrasonic Instrument Co., Ltd., Jiangsu, China. Ultrasonic electric power 150 W, working frequency 40 kHz); DHG-9023A electric heating constant temperature blast drying oven (Shanghai Jinghong Experimental Equipment Co., Ltd., Shanghai, China); FB224 automatic internal calibration electronic analytical balance (Shanghai Sunny Hengping Scientific Instrument Co., Ltd., Shanghai, China); and DF-101S-type heat-collecting constant temperature heating magnetic stirrer (Gongyi Yuhua Instrument Co., Ltd., Shanghai, China) were used.

### Reagent

Ti_3_AlC_2_ (325 mesh, 99%, Forsman Scientific (Beijing) Co., Ltd., Beijing, China); LiF (AR, Shanghai McLean Biochemical Technology Co., Ltd., Shanghai, China); HCl (Shantou Xilong Technology Co., Ltd., Shantou, China); H_2_O_2_ (Sichuan Xilong scientific Co., Ltd., Sichuan, China); lead sulfate (Sinopharm chemical reagent group co., Ltd., Shanghai, China); ethanol (AR, Sichuan Xilong scientific Co., Ltd., Sichuan, China); and 0.5 mmol/L TMB (storage: 2-8°C, Shanghai McLean Biochemical Technology Co., Ltd. Shanghai, China) were used as reagents: 0.012 g of TMB was weighed into a volumetric flask, 100 mL of ethanol:water solution at a ratio of 1:1 was added to the flask, dissolved, and diluted to volume under ultrasonic conditions; 0.1 mmol/L H_2_O_2_ reagent: 100 μL of 30% H_2_O_2_ was taken and transferred into a 10-mL centrifuge tube, diluted with water to 10 mL to obtain the concentration of 0.1 mol/L, and gradually diluted it with water to 0.1 mmol/L; Pb^2+^ aptamer (Apt_Pb_) had a base sequence of 5'-GGTTGGTGTGGTTGG-3' (Shanghai Sangon Biotech Co., Ltd., Shanghai, China); 100 nmol/L Apt_Pb_: Apt_Pb_ was put into a centrifuge at 100 revolutions per minute (rpm), centrifuged for 10 min, 698 μL of deionized water was added to a concentration of 100 μmol/L, and diluted to 100 nmol/L with water when using; Pb^2+^ stock solution: 0.331 g of lead sulfate was accurately weighed and then diluted with water to 10 ml to obtain the concentration of 0.1 mol/L and again diluted to 1 × 10^−8^ mol/L with water.

### Preparation of MXenes

In a 50-mL clean conical flask, 0.9 g LiF and 20 mL 9 mol/L HCl were added and stirred at 500 rpm for 30 min; then, 0.3 ± 0.01 g Ti_3_AlC_2_ was accurately weighed and slowly added. It was transferred to a polytetrafluoroethylene tank and heated in a microwave heater at 160°C for 2 h. After cooling, it was put into a centrifuge to run at 5,000 rpm for 5 min, then washed with deionized water (5 × 20 mL, the pH ~6), and finally washed with ethanol (2 × 10 mL). The precipitate was dried in an oven at 70°C. Approximately, 20 mg MXene was accurately taken to a 10-mL volume, then 100 μL of this solution was taken to dilute to 10 mL to obtain 20 mg/L MXene nanosol.

### Experimental method

In a 5-mL graduated test tube with a stopper, 200 μL 20 mg/L MXene solution and 200 μL 100 nmol/L Apt_Pb_ were first added, and then, 0-400 μL 10^−8^ mol/L Pb^2+^ was added and allowed to stand for 10 min, and next, 100 μL 0.5 mmol/L TMB solution, 135 μL 0.1 mmol/L H_2_O_2_ solution, 120 μL Tris-HCl buffer solution were added. After dilution to 2 mL, it was heated in a water bath at 45°C for 30 min and then put in ice water to stop the reaction. The fluorescence spectrum was obtained by scanning with a fluorescence spectrophotometer. The fluorescence peak was measured at 415 nm as F, and no Pb^2+^ solution was taken as blank F_0_. ΔF = F-F_0_ value was calculated.

## Results and discussion

### Analysis principle

**Figure 1:**
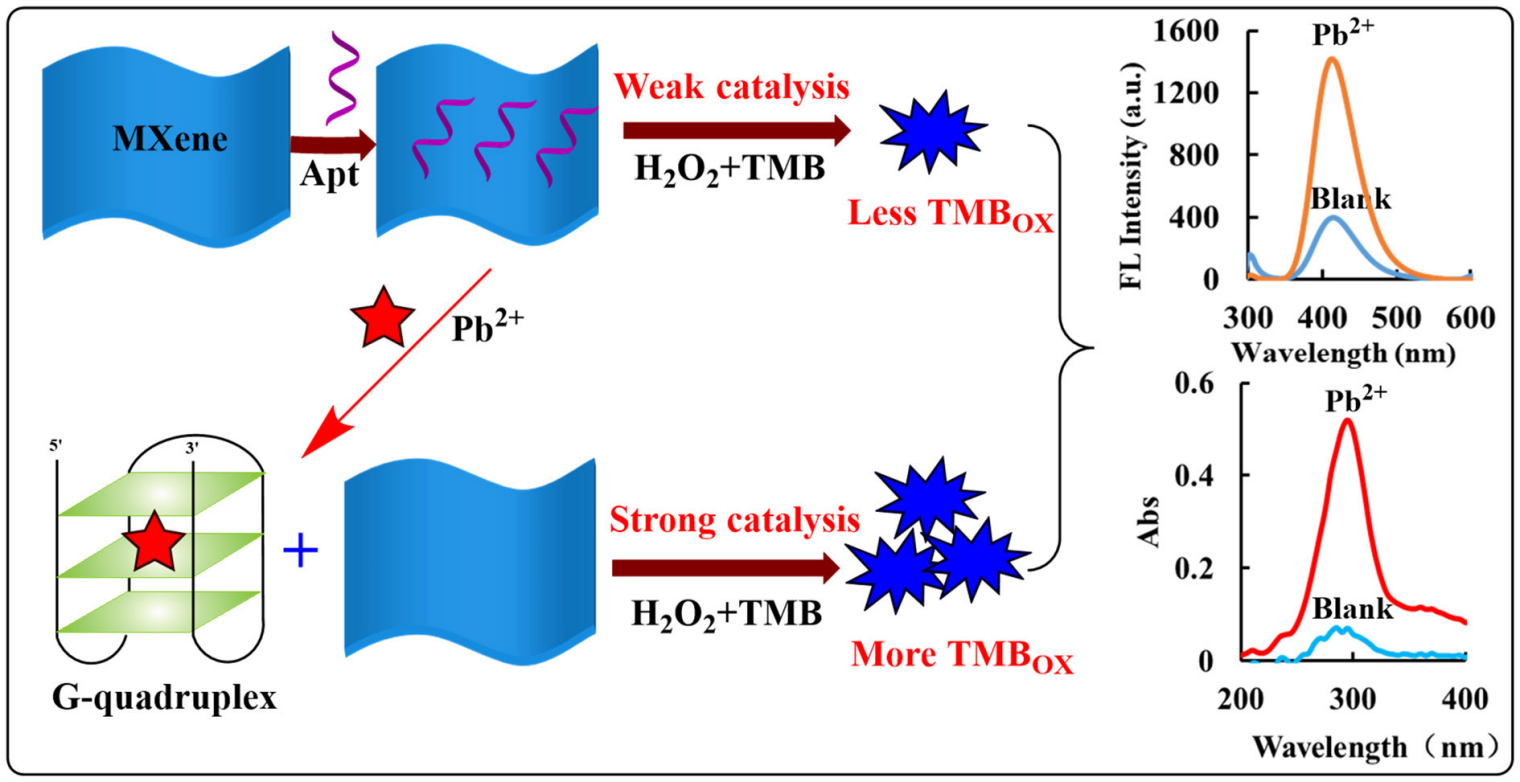
Analysis principle of fluorescence/Abs dimode detection of Pb^2+^ based on Apt_Pb_-Pb^2+^-MXene-H_2_O_2_-TMB.

MXene etching environment causes Ti on its surface to coordinate with polar groups such as -O and -OH after exposure, so it has better electronegativity and hydrophilicity, which are beneficial to disperse in the aqueous phase and form a relatively stable dispersing sol (24). In pH 3.21 Tris-HCl buffer solution, MXene has a strong catalytic effect on H_2_O_2_ oxidation of TMB to form TMB oxidation products (TMB_OX_). Its strong catalytic performance may originate from its layered structure, which can expose more active sites to contact with reactants. At the same time, it has more surface-free electrons, and the synergistic effect of the two makes MXene to have better catalytic activity. As a hydrophilic molecule, Apt can encapsulate MXene through hydrogen bonding, Howard force, electrostatic adsorption, etc., decreasing its active site and weakening the catalytic activity of MXene. After the addition of the target Pb^2+^, Pb^2+^ specifically binds to the nucleic acid Apt, and MXene is released at this time, and its catalytic activity is restored. With the increase of Pb^2+^ concentration, the more MXene desorbed, the faster the catalytic reaction of H_2_O_2_ oxidation of TMB; the increase of the generated TMB_OX_ concentration and the generated fluorescence and absorption signals have a linear relationship with added Pb^2+^ concentration. Accordingly, a fast, simple, and highly sensitive fluorescence/absorption dimode sensing platform for the determination of Pb^2+^ is established (Figure 1).

### Characterization of nanomaterials

**Figure 2:**
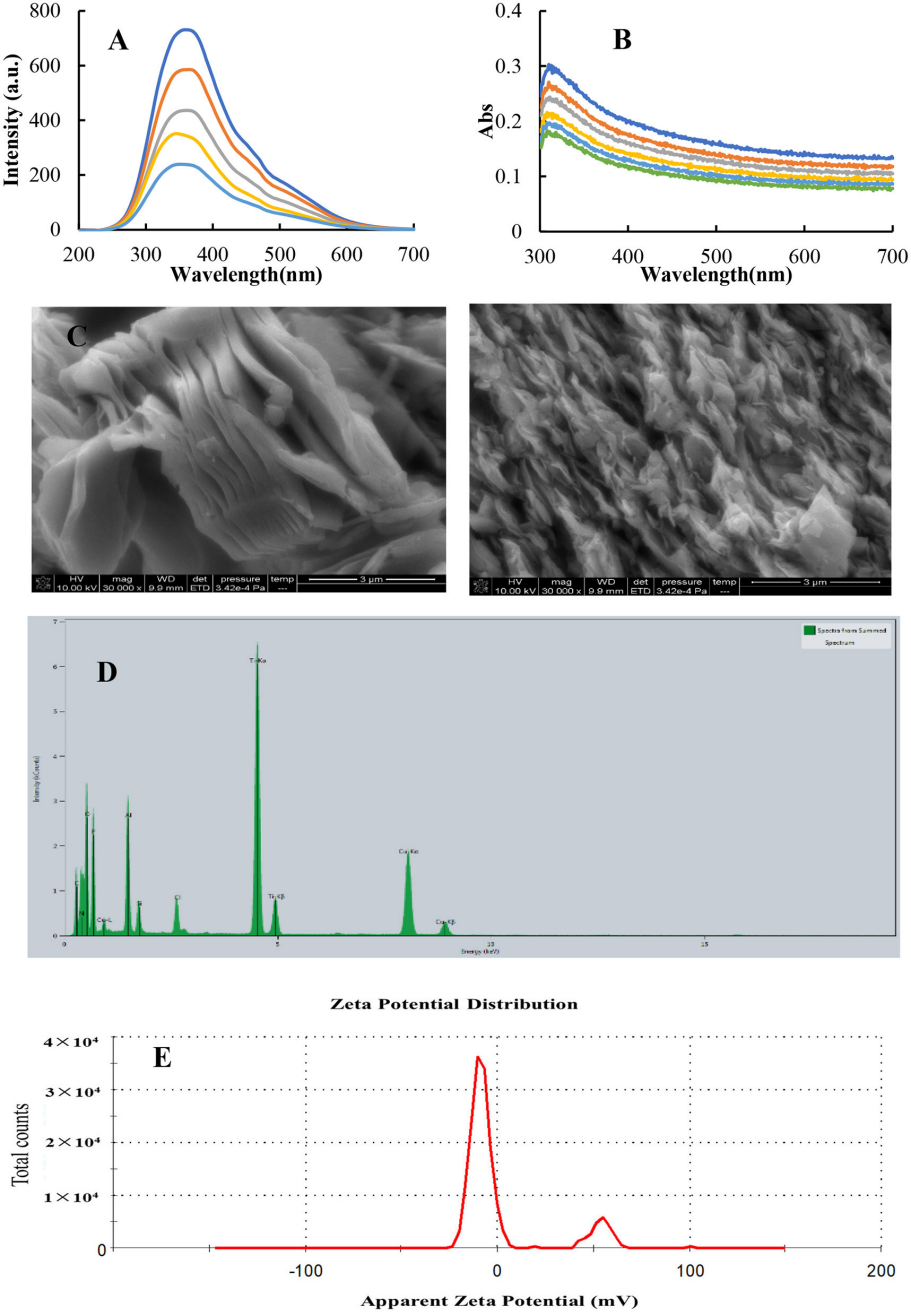
Molecular spectra, SEM, and surface Zeta charge of MXene. **(A)** RRS spectrum, (a-e: 3.6, 4.4, 8.0, 10.5, and 13.3 mg/L) MXene. **(B)** Abs spectrum, (a-g: 13.3, 14.3, 15.4, 16.7, 18.2, and 20 mg/L) MXene. **(C)** SEM images of MXene. **(D)** EDS spectrum of MXene. **(E)** Surface zeta charge of 10 mg/L MXene nanosol.

Resonance Rayleigh scattering (25, 26) was a simple and sensitive molecular spectroscopy to study nanoparticles, and Abs spectrometry was a simple and low-cost spectral technique. We investigated the properties of MXenes using these two spectroscopic techniques. The RRS spectrum of MXenes produces a characteristic RRS peak at 360 nm (Figure 2A), and the intensity of the characteristic peak gradually increases with an increase in the concentration of MXene. This is because the concentration of MXnen increases, thus increasing the number of nanoparticles in the system and hence enhancing RRS intensity. RRS spectra were electron spectra of MXene nanoparticles with a broad band. The surface electrons of MXene nanoparticles were irradiated by the incident photons from the light source to transit in the excitation state. Electrons in the excitation state were unstable and returned from the excitation state to the ground state to emit Rayleigh scattering photons. The incident photons change continuously, and its scattering photons are also continuous. It can be seen from the Abs spectra of MXene (Figure 2B) that the ultraviolet Abs peak of MXene is at 313 nm, and its peak intensity increases with the increase in the concentration of MXene; this Abs may correspond to the band gap energy of oxidized MXene (27). The morphology of MXene solid was investigated by scanning electron microscopy (SEM). The MXene material is thinner and has a distinct layered structure from an edge (Figure 2C), which can expose more surfaces, provide more sites for active components, and increase contact between the catalyst and the reaction system. C, N, O, and Ti elements can be observed in the energy dispersive spectroscopy (EDS) images of MXene samples (Figure 2D), and the corresponding energy peaks of C, N, and O elements are 0.525, 0.392, and 0.277 KeV, respectively. The energy peaks of Ti are at 4.931 and 0.530 KeV. However, the layered structure of MXene can expose more active sites and increase the contact with the reactants, thereby accelerating the reaction. In this study, zeta potential analyzer was used to accurately measure the surface Zeta charge of nanoparticles. The surface zeta potential of MXene is −16.5 mV (Figure 2E), which is favorable for the system to form a dispersed nanosol. At the same time, it shows that the surface had more surface-free electrons, which could accelerate the electron transfer from the TMB-H_2_O_2_ reaction. It could be seen that MXene NSs provided a fast electron transfer pathway in the catalytic reaction.

### Fluorescence spectra of nanocatalysis and analysis system

**Figure 3:**
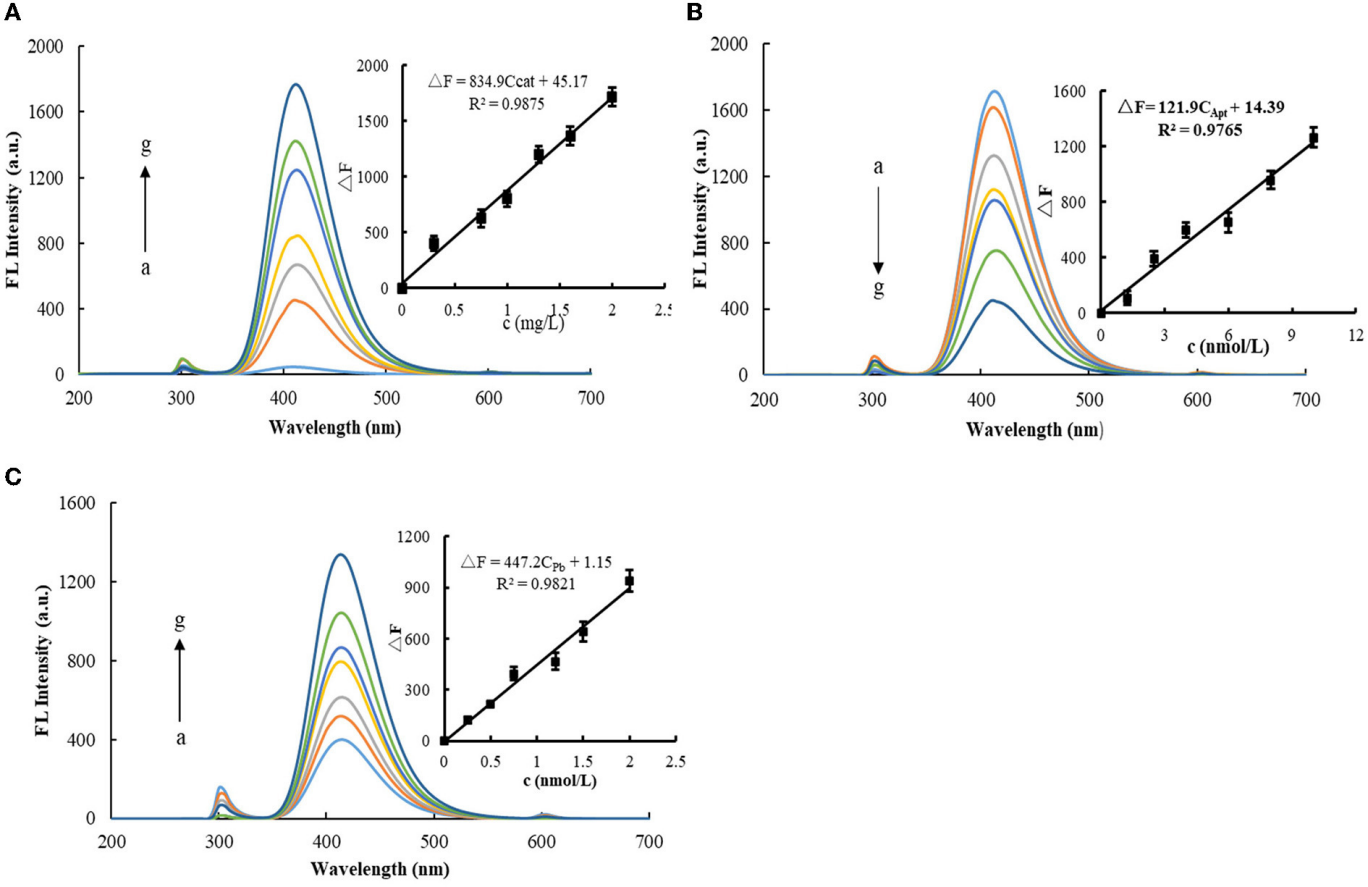
Fluorescence spectra of MXene nano-catalyzed H_2_O_2_-TMB system. **(A)** 2.5 × 10^−2^ mmol/L TMB + 6.8 × 10^−3^ mmol/L H_2_O_2_ + 120 μL Tris-HCl (pH = 3.31) + (a-g: 0, 0.3, 0.75, 1.0, 1.3, 1.6, and 2.0 mg/L) MXene. **(B)** 2.5 × 10^−2^ mmol/L TMB + 6.8 × 10^−3^ mmol/L H_2_O_2_ + 120 μL Tris-HCl (pH=3.31) + (a-g: 0, 1.25, 2.5, 4.0, 6.0, 8.0, and 10 nmol/L) Apt_Pb_. **(C)** 10 nmol/L Apt_Pb_ + 2.0 mg/L MXene +2.5 × 10^−2^ mmol/L TMB + 6.8 × 10^−3^ mmol/L H_2_O_2_ + 120 μL Tris-HCl (pH = 3.31) + (a-g: 0, 0.25, 0.5, 0.75, 1.2, 1.5, and 2.0 nmol/L) Pb^2+^.

Figure 3A shows the fluorescence spectra of the H_2_O_2_-TMB-MXene catalytic system. There is a strong fluorescence peak at 415 nm. In addition, a small peak at 300 nm was observed, which was a Rayleigh scattering peak due to MXene nanoparticles. When MXene nanosol was not added, the fluorescence signal generated *via* the system was low. After adding MXene nanosol, the fluorescence signal of the system was gradually enhanced. It was proven that MXene had a catalytic effect on H_2_O_2_-TMB, and the fluorescence signal was positively correlated with the concentration of MXene nanosol. After selecting a suitable amount of MXene concentration and then adding Apt (Figure 3B), Apt could coat MXene and reduce its catalytic performance. Therefore, with the increase of Apt, the catalytic effect of MXene was gradually weakened, and the fluorescence signal generated by the system was gradually decreased. Figure 3C shows the fluorescence spectra of the Pb^2+^-Apt_Pb_-MXene-H_2_O_2_-TMB system. When Pb^2+^ was added, Pb^2+^ and Apt specifically recognize and release free MXene, thereby restoring the catalytic effect of the system. When the concentration of Pb^2+^ gradually increased, the more the MXene nanoparticles released by the Pb^2+^-Apt complex from the surface of MXene, the stronger the catalytic activity, and the fluorescence signal of the system increased linearly with the concentration of Pb^2+^.

### Abs spectra of nanocatalytic and analytical systems

**Figure 4:**
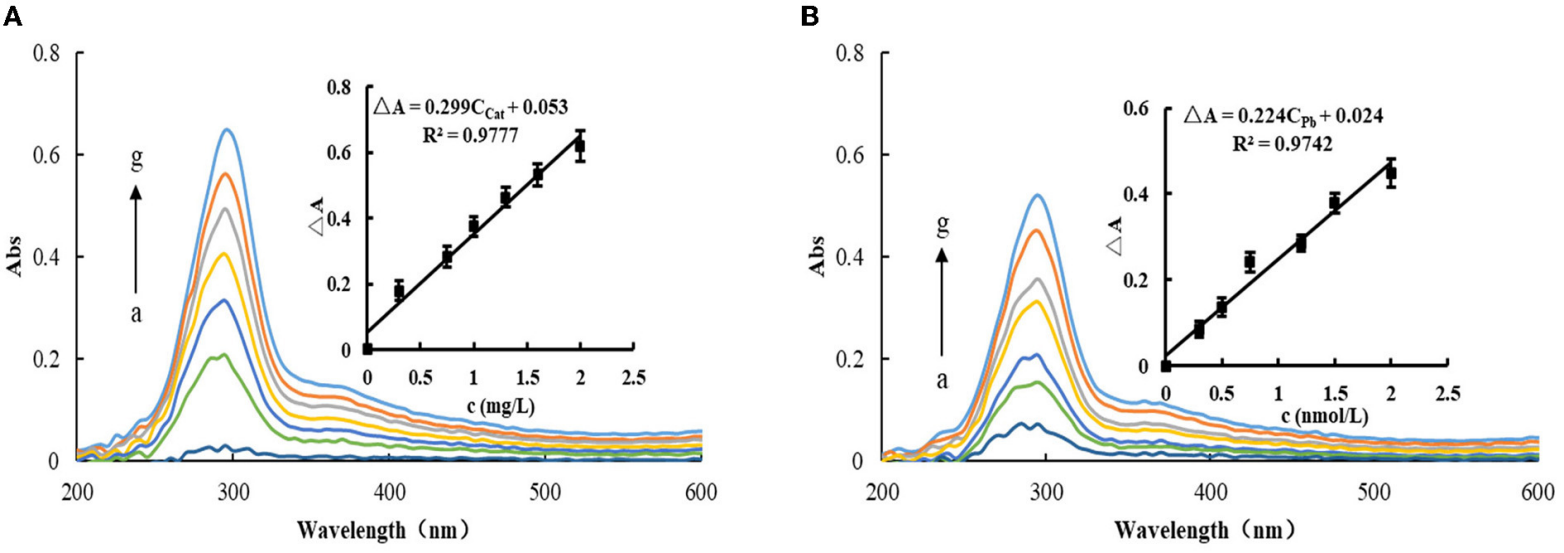
Abs spectra of the MXene nanocatalytic system. **(A)** 2.5 × 10^−2^ mmol/L TMB + 6.8 × 10^−3^ mmol/L H_2_O_2_ + 120 μL Tris-HCl (pH = 3.31) + (a-g:0, 0.3, 0.75, 1.0, 1.3, 1.6, and 2.0 mg/L) MXene. **(B)** 10 nmol/L Apt_Pb_ + 2.0 mg/L MXene + 2.5 × 10^−2^ mmol/L TMB + 6.8 × 10^−3^ mmol/L H_2_O_2_ + 120 μL Tris-HCl (pH = 3.31) + (a-g: 0, 0.3, 0.5, 0.75, 1.2, 1.5, and 2.0 nmol/L) Pb^2+^.

Under selected experimental conditions, the reaction of TMB-H_2_O_2_ was slow, and the system produced less TMB_OX_. After adding MXene nanosol, due to its catalytic effect, the system gradually changed from colorless to dark blue, which was studied *via* an Abs meter. The results showed that the generated TMB_OX_ produces an Abs peak at 295 nm, and the corresponding Abs signal at 295 nm also increases with the increase of MXene nanosol concentration (Figure 4A). When Apt_Pb_ was added to the catalytic system, it was adsorbed and wrapped on the surface of MXene, resulting in the reduction of its active sites and weakening of the catalytic activity. Therefore, the system generated less TMBox and the Abs signal weakened. When the target Pb^2+^ was added, it binds specifically with Apt and falls off from the surface of MXene and released free MXene, which restored its catalytic activity and enhanced the Abs signal. With the increase of Pb^2+^ concentration, the released MXene nanoparticles increase and the change of Abs of the system increases linearly with the increase of Pb^2+^ concentration (Figure 4B). Although Abs could also be used for its determination, the sensitivity was lower than the fluorescence assay, especially since the slope of the working curve was smaller than that of the fluorescence method. Therefore, the fluorescence method was chosen for the determination of Pb^2+^.

### MXene catalytic mechanism and Apt inhibition

Under selected conditions, the oxidation rate of TMB *via* H_2_O_2_ was very slow, less TMB_OX_ was generated, and the fluorescence signal was very weak. As a catalyst, MXene nanosol had more surface free electrons and a layered interlayer structure, and the coordination between the two enhanced its catalytic activity, which was conducive to the decomposition of H_2_O_2_ into hydroxyl radicals and accelerated the electron transfer of the reaction of H_2_O_2_ oxidation TMB, thereby rapidly generating TMB_OX_ and producing a strong fluorescence signal.

With the increase of nanocatalyst concentration, the catalytic ability was enhanced, and the TMB_OX_ generated *via* the catalyst increased, and the fluorescence intensity of the system had a certain linear relationship with the catalyst concentration. When Apt was added to the system, Apt was attached to the surface of the catalyst, reducing its active site and blocking the contact between the catalyst and the reactant, thereby inhibiting its catalytic activity. With the increase in the amount of Apt added, the catalytic fluorescence signal weakened, and ΔF_415nm_ of the system was negatively correlated with the added concentration of Apt. After adding Pb^2+^, it was combined with Apt to release MXene and restore its catalysis, which led to the recovery of fluorescence in the system. The main reactions were as follows:


$$\begin{array}{l} \text{ MXene}+{\text{H}}_{2}{\text{O}}_{2}\to \text{·OH}\\ \text{MXene}+{\text{H}}_{2}{\text{O}}_{2}\to \text{H}{\text{O}}_{2}\cdot \\ \text{ ·OH}+\text{TMB}\to \text{TM}{\text{B}}_{\text{OX}}\\ \text{ H}{\text{O}}_{2}\text{·}+\text{TMB}\to \text{TM}{\text{B}}_{\text{OX}} \end{array}$$

### Selection of analysis conditions

According to the experimental method, the reaction conditions of the Apt-Pb-MXene-H_2_O_2_-TMB-(Tris-HCI) system were optimized *via* the single-variable method. It was found that, when the pH of the Apt-Pb-MXene-H_2_O_2_-TMB-(Tris-HCI) system was optimized, the pH is optimized *via* changing the amount of HCl, and ΔF_415nm_ of the system reached the maximum value at pH = 3.31 (Supplementary Figure S1A). The concentration of H_2_O_2_ was optimized, when the concentration of H_2_O_2_ in the system was 6.8 × 10^−3^ mol/L and ΔF_415nm_ of the system reached the maximum value (Supplementary Figure S1B). TMB has no fluorescence, and the experiment found that the oxidation product of TMB had fluorescence. When the concentration of TMB was 2.5 × 10^−2^ mmol/L, ΔF_415nm_ of the system reached the maximum value (Supplementary Figure S1C), so the concentration of TMB is selected to be 2.5 × 10^−2^ mmol/L. The concentration of MXene was optimized when the concentration of MXene in the system is 2.0 mg/L and ΔF_415nm_ of the system reached the maximum value (Supplementary Figure S1D). When the Apt_pb_ concentration is 10 nmol/L, ΔF_415nm_ of the system reached the maximum value (Supplementary Figure S1E). All the above-optimized conditions were carried out in a water bath at 45°C, and the reaction time was 30 min. Under different temperature conditions, the reaction of the system was different. When the temperature of the water bath was 45°C, ΔF_415nm_ of the system reached the maximum value (Supplementary Figure S1F), so the reaction temperature was selected as 45°C. Since when the reaction time was 30 min, ΔF_415nm_ of the system reached the maximum value, so the reaction time was optimized (Supplementary Figure S1G) and selected as 30 min.

### Effects of interfering ions

The effect of coexisting substances in the Apt-Pb-MXene-H_2_O_2_-TMB fluorescence system on the determination of 0.25 nmol/L Pb^2+^ was investigated according to the experimental method. Experimental results show that the relative error is within ±10%, 1,000 times of K^+^, Ca^2+^, Al^3+^, Mg^2+^, Zn^2+^, ${\text{CO}}_{3}^{2-}$, Cu^2+^, and ${\text{NH}}_{4}^{+}$, 500 times of Na^+^, Cr^3+^, S_2_${\text{O}}_{3}^{2-}$, and Cl^−^, 100 times of Ba^2+^, Fe^2+^, ${\text{SO}}_{4}^{2-}$, NO^2−^, and ${\text{PO}}_{4}^{3-}$, 50 times of Cr^6+^, Co^2+^, and Hg^2+^, which do not interfere with the determination of Pb^2+^ (Supplementary Table S1), indicating that the method has good selectivity.

### Working curves

Under optimal conditions, the Apt-Pb-MXene-H_2_O_2_-TMB system was used to detect Pb^2+^ *via* fluorescence/Abs. According to the concentrations of Pb^2+^ and the corresponding ΔF/ΔA values, the working curves were plotted. In a fluorescence assay, the linear regression equation is ΔF = 447.2C_Pb_+1.15, the LR is 5.0 × 10^−2^-2.0 nmol/L, and the coefficient of 0.9821. For the Abs method, the linear regression equation is ΔA = 0.224C_Pb_ + 0.024, the LR is 0.3-2.0 nmol/L, and the coefficient is 0.9742. The standard blank was measured for 11 consecutive times of fluorescence intensity, and the standard deviation (SD) was calculated as S_FL_ = 5.48, S_Abs_ = 5.5 × 10^−3^. According to the detection limit formula, DL = 3S/k (k is the slope of the standard curve), the calculated detection limit of the fluorescence method is 3.7 × 10^−2^ nmol/L, and the detection limit of Abs is 0.1 nmol/L. Compared with the reported detection methods (27–31), the fluorescence assay is simpler and faster (Table 1).

**Table 1:** Comparison with the reported analysis methods for Pb^2+^.

**Method**	**Analysis principle**	**LR**	**DL**	**Comments**	**Ref**.
Colorimetry	Pb^2+^ accelerated the dissolution rate of AuNRs induced by sodium thiosulfate, resulting in the blueshift and attenuation of the absorption band of AuNRs and the apparent irreversible color change of gold colloids from blue to red.	25~300 nM	20 nM	Good selectivity but low sensitivity, complicated operation.	(28)
ECL	RNA cleavage activity was activated by Pb^2+^, and its substrate strand is cleaved, resulting in the release of Ru(bpy)(dppz) from the DNA membrane, accompanied by a decrease in photocurrent.	0.5~20 nM	0.1 nM	High sensitivity and selectivity but complicated operation.	(29)
Abs	The addition of Pb^2+^ creates a complex with a color change visible to the naked eye.	-	40 nM	Good selectivity, but low sensitivity.	(30)
SERS	When Pb^2+^ ions bind to the substrate, AuNPs conjugates will be cleaved from the gold surface, resulting in the Raman signal decreasing.	-	1.0 nM	Sensitive and accurate, but complicated and time-consuming.	(31)
Fluorescence	The fluorescence of FAM was quenched by AuNFs due to fluorescence resonance energy transfer. After the addition of Pb^2+^, Apt was transformed into a G-quadruplex structure, which resulted in a fluorescence increase.	0.5 nM~1 μM	0.285 nM	High sensitivity and good selectivity, but the process is complicated	(32)
Nanocatalytic fluorescence	The MXene catalysis was regulated by the Apt, and the fluorescent signal of the generated TMB_OX_ was recorded to detect Pb^2+^.	0.05~2 nM	0.037 nM	High sensitivity, good selectivity, and simple operation.	This method

### Analysis of samples

Accurately, 10 mL of milk or wastewater was transferred to a 100 mL porcelain crucible, 10 mL of 1:1 nitric acid was added, heated with a low fire, and carbonized without smoke, milk or wastewater was transferred to a high-temperature electric furnace and burned to a constant weight at about 700°C, and a crucible was taken out after the sample turned into white ash. After cooling, 5 mL of 1:1 nitric acid was added and heated at a low temperature to dissolve the ash. After removing all nitrogen oxides, 10 mL of water was added to the beaker, heated continuously for 2-3 min, filtered with quantitative filter paper, and washed with hot water. The washing solution was incorporated into a 25-mL volumetric flask, diluted with water to the scale, and shaken well for standby. The APt-Pb^2+^-MXene-H_2_O_2_-TMB fluorescence analytical system was selected for the detection of Pb^2+^, and a standard concentration of Pb^2+^ was added for the measurement of recovery. The test results are given in Table 2. The results are in agreement with the atomic absorption spectrometry (AAS), the relative deviation (RSD) is between 2.4 and 5.4%, and the recovery rate is between 92.4 and 96.5%.

**Table 2:** Analytical results of Pb in milk and wastewater samples.

**Sample**	**Average**	**Added**	**Found**	**RSD**	**Recovery**	**Content**	**AAS results**
	**(nmol/L, *n* = 5)**	**(nmol/L)**	**(nmol/L)**	**(%)**	**(%)**	**(nmol/L)**	**(nmol/L)**
Milk 1	0.416	0.375	0.351	5.2	93.6	93.2	92.2
Milk 2	0.450	0.375	0.346	4.7	92.4	90.0	93.0
Milk 3	0.436	0.375	0.356	5.0	95.1	87.2	85.2
Milk 4	0.470	0.375	0.362	4.9	96.5	94.0	91.0
Wastewater 1	0.450	0.375	0.801	5.4	93.6	90.0	86.5
Wastewater 2	0.512	0.375	0.868	4.8	94.8	101	98.0
Wastewater 3	0.640	0.375	0.996	2.4	95.0	128	136

## Conclusion

This study is based on the catalysis of H_2_O_2_-TMB using MXene. Under the catalysis of MXene NSs, the system generated TMB_OX_ with a fluorescent and Abs signal. The binding of Apt_Pb_ to MXene inhibited its catalytic effect. When Pb^2+^ was added, Pb^2+^ could form a very stable complex with Apt, thus releasing MXene and restoring its catalytic ability, resulting in a linear enhancement of the fluorescence and Abs signal of the system. Accordingly, a dimode molecular spectral analysis platform was established for the detection of Pb^2+^, based on MXene-H_2_O_2_-TMB fluorescence/Abs indicator reaction and specific Apt reaction. The dimode analytical platform had the advantages of high sensitivity, simple and fast methods, and so on. Although this assay was sensitive, the precision needs to be improved. In the future, other highly sensitive techniques, such as SERS, will be used to study the nanocatalytic indicator reaction to develop a highly sensitive method and a multimode method.

## Data availability statement

The original contributions presented in the study are included in the article/Supplementary material, further inquiries can be directed to the corresponding author.

## Author contributions

SZ: software, formal analysis, investigation, data curation, and writing—original draft preparation. QW: investigation, data curation, and writing—original draft preparation. CZ: software, data curation, and writing—original draft preparation. CY: writing—original draft preparation. CL: validation, resources, visualization, project administration, and funding acquisition. ZJ: conceptualization, methodology, writing—review and editing, and supervision. All authors contributed to the article and approved the submitted version.

## Funding

This work was supported by the Natural Science Foundation of Guangxi (No. 2022GXNSFDA035073) and the National Natural Science Foundation of China (No. 21767004).

## Conflict of interest

The authors declare that the research was conducted in the absence of any commercial or financial relationships that could be construed as a potential conflict of interest.

## Publisher's note

All claims expressed in this article are solely those of the authors and do not necessarily represent those of their affiliated organizations, or those of the publisher, the editors and the reviewers. Any product that may be evaluated in this article, or claim that may be made by its manufacturer, is not guaranteed or endorsed by the publisher.
