# Supplementary material for: MXene catalytic amplification-fluorescence/absorption dimode aptamer sensor for the detection of trace Pb2+ in milk
Source: Front Nutr. 2022 Oct 18;9:1008620. doi: 10.3389/fnut.2022.1008620 (PMC9622933; doi:10.3389/fnut.2022.1008620)
Supplement: Supplementary file 1 [file Data_Sheet_1.DOCX]

**MXene catalytic amplification-fluorescence/absorption dimode aptamer sensor for detection of trace Pb^2+^ in milk**

**Shengfu Zhi**^1,2^**, Qi Wei**^1,2^**, Chi Zhang**^2^**, Chengguang Yi**^2^**, Chongning Li**^1,2*^**, Zhiliang Jiang**^2*^

^1^ School of Public Health, Guilin Medical University, Guilin 541199, China; ^2^Guangxi Key Laboratory of Environmental Pollution Control Theory and Technology, Guilin 541006, China.


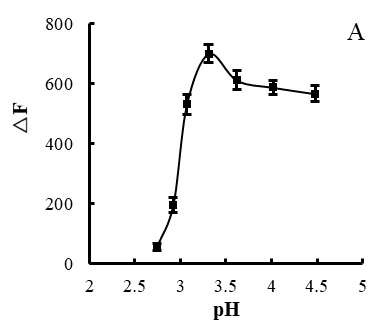

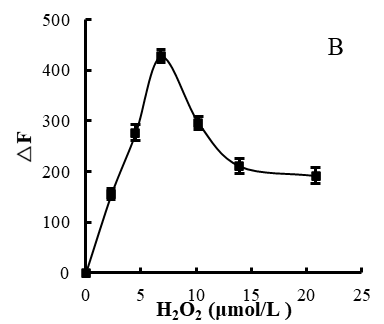


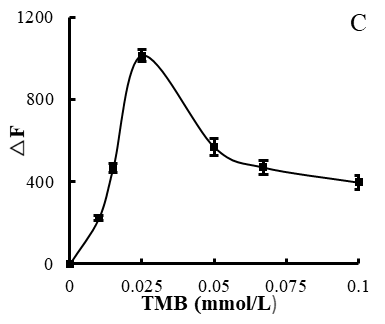

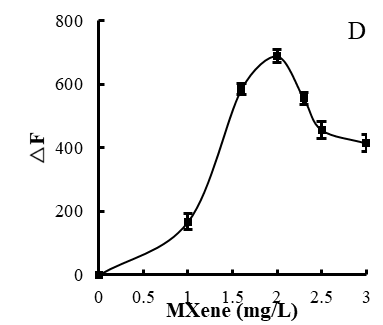


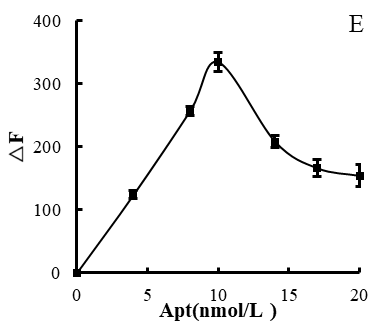

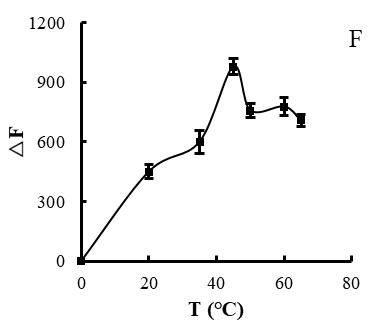


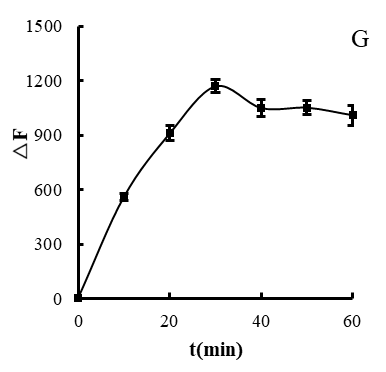


**FIGURE S1.** Selection of Analysis Conditions. **(A)** The effect of pH on the ΔF of the system, 0.25 nmol/L Pb^2+^+10 nmol/L Apt_Pb_ + 2.0 mg/L MXene + 2.5×10^-2^ mmol/L TMB + 6.8×10^-3^ mmol/L H_2_O_2_. **(B)** The effect of H_2_O_2_ on the ΔF of the system, 0.25 nmol/L Pb^2+^+10 nmol/L Apt_Pb_ + 2.0 mg/L MXene + 2.5×10^-2^ mmol/L TMB + 120 μL Tris-HCI (pH=3.31). **(C)** The effect of TMB on the ΔF of the system, 0.25 nmol/L Pb^2+^+10 nmol/L Apt_Pb_ + 2.0 mg/L MXene + 6.8×10^-3^ mmol/L H_2_O_2_ +120 μL Tris-HCI (pH=3.31). **(D)** The effect of MXene on the ΔF of the system, 0.25 nmol/L Pb^2+^ + 10 nmol/L Apt_Pb_ + 2.5×10^-2^ mmol/L TMB + 6.8×10^-3^ mmol/L H_2_O_2_ + 120 μL Tris-HCI (pH=3.31). **(E)** The effect of Apt on the ΔF of the system, 0.25 nmol/L Pb^2+^+ 2.0 mg/L MXene + 2.5×10^-2^ mmol/L TMB + 6.8×10^-3^ mmol/L H_2_O_2_ + 120 μLTris-HCI (pH=3.31). **(F)** The effect of temperature on the ΔF of the system, 0.25 nmol/L Pb^2+^+10 nmol/L Apt_Pb_ + 2.0 mg/L MXene + 2.5×10^-2^ mmol/L TMB + 6.8×10^-3^ mmol/L H_2_O_2_ + 120 μL Tris-HCI (pH=3.31). **(G)** The effect of time on the ΔF of the system, 0.25 nmol/L Pb^2+^+10 nmol/L Apt_Pb_ + 2.0 mg/L MXene + 2.5×10^-2^ mmol/L TMB + 6.8×10^-3^ mmol/L H_2_O_2_ + 120 μL Tris-HCI (pH=3.31).

**TABLE S1.** Effects of interfering ions on the fluorescence assay

| **Coexisting substance** | **Relative multiple** | **Relative error**  **（%）** | **Coexisting**  **substance** | **Relative multiple** | **Relative error**  **（%）** |
| --- | --- | --- | --- | --- | --- |
| K^+^ | 1000 | 5.5 | Cu^2+^ | 1000 | 7.2 |
| Ca^2+^ | 1000 | 6.2 | Co^2+^ | 50 | 0.94 |
| Na^+^ | 500 | 8.0 | Ba^2+^ | 100 | 6.2 |
| Mg^2+^ | 1000 | -4.2 | Cl^-^ | 500 | 4.7 |
| Al^3+^ | 1000 | 9.2 | SO_4_^2-^ | 100 | 5.9 |
| Zn^2+^ | 1000 | -1.1 | NO_2_^-^ | 100 | 4.2 |
| Fe^2+^ | 100 | -2.7 | S_2_O_3_^2-^ | 500 | 7.9 |
| NH^4+^ | 1000 | 4.6 | CO_3_^2-^ | 1000 | 6.2 |
| Cr^3+^ | 500 | 3.7 | PO_4_^3-^ | 100 | 3.1 |
| Cr^6+^ | 50 | -1.9 | Hg^2+^ | 50 | -8.4 |
